# Evaluation metrics in science: current status and prospects

**DOI:** 10.1590/1518-8345.0000.2865

**Published:** 2017-06-05

**Authors:** Lilian Nassi-Calò

**Affiliations:** Coordinator of Scientific Communication in Health at BIREME/PAHO/WHO and a collaborator of SciELO. E-mail: calolili@paho.org

**Figure:**
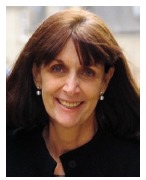

The evaluation of science uses a variety of bibliometric indicators mostly based on citations despite not having an unequivocal relationship between citations and scientific quality. These indicators however, encompass more than an indication of visibility, relevance and impact of the articles and may represent in a researcher's career prestige, job opportunities, career promotion, awards, reserach grants and other rewards. 

## Indicators of scientific impact

One of the first and most widely used indexes ever created, the Impact Factor (IF), dates from 1975, when Eugene Garfield, the founder of the Institute for Scientific Information - ISI, introduced it to support the selection of subscription journals by libraries^1^. Along the way, from this start to its ubiquitous use to rank publications, researchers and institutions, its characteristics and peculiarities were neglected for the sake of convenience of an index that was easy to calculate and widely disseminated by all areas of knowledge worldwide. Until mid- 2016, the Journal Citation Reports (JCR) database, which publishes the IF, and the Web of Science belonged to the Thomson Reuters Company, and now iintegrate the products of Clarivate Analytics. 

The IF had no serious competition until 2004, when multinational publisher Elsevier created the Scopus bibliographic database, and from it, the SCImago Journal & Country Rank (SJR) index was launched in 2008, available in open access, unlike JCR, which requires a subscription. The form of calculating the SJR and IF present a few difference, but both basically appraise citations per time interval and have a linear relationship. 

In 2005, physicist Jorge E. Hirsch from the University of California, San Diego created the h index to measure not only the impact, but also the productivity of researchers. This indicator gained popularity quickly and is also applied to journals or institutions, often being referred in *curricula vitae*, such as the Lattes Platform. 

Additionally, there are indexes such as *Eigenfactor* and *Article Influence* ^2^, also citation-based and whose calculations use elegant algorithms and are available in open access, are not frequently used or mentioned.

Despite being widely used in science evaluation processes, the limitation and precariousness of the use of citation indicators are recognized by the global scientific community, given the peculiarities and biases of the citation indicators used to measure the performance of articles, journals, researchers, institutions and countries. Initiatives that aim to curb or discourage their use such as the San Francisco Declaration on Research Assessment^3^ (DORA) or the Leiden Manifesto^4^ are supported by researchers and institutions around the world. These initiatives are supported by scientific societies, universities, funding agencies and journals, among others^5^.

Understanding the nature of the indicators, how they are calculated, their applicability and limits is essential not only for specialists in scientometrics and funding agency technicians, but for the entire scientific community. After all, the researchers themselves evaluate their peers in hiring and career progression processes; therefore, it is advisable to go beyond the simple analysis of publication numbers, journal IF or their h index. Associate Philosophy of Science Professor at UFRJ Antonio Augusto P. Vieira's consideration is eloquent: "The fact that the use of an indicator makes one author or the other eligible due to the fact that he/she has published in a journal with a higher IF, should be surprising, since more importance is given to where he/she was published than the reading of his/her work"^6^. All those involved in the evaluation of science and in reward systems must be committed to this concept so as not to infer snap judgments or be unjust. 

One of the criticisms made to bibliometric indices based on citations is because the practice of citing articles is extremely complex and influenced by countless factors. Thus, the true reasons for citing one article and not another has nothing to do with quality, validity or relevance of the studies^7^. In fact, it was not possible to establish a relationship about the most cited study and his/her best study in researcher's self-evaluation^8^. Standardizing citation metrics might level the indices per area of knowledge, publication age, type of document and comprehensiveness of the database in which they were recorded, thus allowing for better balanced comparisons in evaluation processes^9^. 

## Alternative metrics or altmetrics

Social media are very efficient for the sharing of news, opinions and content in general. More recently, they have been widely used as science evaluation metrics and are called altmetrics or "alternative metrics"^10^.

Studies estimate that only taking formal citations into consideration, we will be disregarding almost 50% of the scientific literature published worldwide. Altmetrics^11^ have been gaining credibility in the evaluation of publications and researchers. The *Altmetric* index monitors various social networks in the sharing of scientific articles: blogs, *Twitter, Facebook, Mendeley, YouTube, ResearchGate, Google, Reddit, LinkedIn*, print and online news, mention in the elaboration of public policies, and others. One study^12^ shows that altmetrics present a correlation with impact indices based on citations and can be used to complement them along with peer review and usage measures such as access and download. 

Like any new concept, it often generates doubts and questions about its legitimacy, especially due to the fact it uses 'informal' tools to measure the impact of science, which is essentially formal. It is possible that the skepticism of the academic community towards altmetrics is comparable to the reaction caused by the use of the Internet in the 1990's as a platform to publish scientific journals.

It is important to consider new forms of scientific communication, which are already influencing how research results are being published, disseminated and evaluated. These are preprint electronic repositories. The first one is arXiv^13^, created in 1991 to publish preliminary versions of articles in the areas of physics, astronomy, computer sciences and statistics. The authors post their articles before formally submitting them to a journal, in order to receive comments from the scientific community and to ensure the authorship of an idea or research result. Many articles, however, don't get formally published, not because of lack of relevance or quality, but because publishing in the repository *per se* is academically recognized, at least in the area of physics, with the same weight as a journal article. Comments are posted online and the authors can update their articles based on this post-publication peer review. Based on the success of arXiv, preprint repositories for different disciplines are being created. BioRxiv was launched in 2013 for the life sciences, and in February of 2017, it had over 8,000 preprints. Preprint repositories in the areas of chemistry (ChemRxiv), psychology (PsyArXiv) and social sciences (SocArXiv) are in the process of being implemented. The academic community's incentive and recognition of this form of publishing is demonstrated by initiatives such as ASAP Bio^14^, which encourages the publishing of preprints and post-publication peer review, in addition to the growing adoption and recognition of preprint repositories by institutions, international organizations and funding agencies^15^. This is a form of publishing particularly suited for the fast and open dissemination of results, as required in cases of public health emergencies, such as the recent Ebola and Zika epidemics.

Scientific communication as we know is rapidly evolving for the better, I believe, with more agility, transparency, responsibility, improved access and the use of research for the benefit of individuals and society, and we must follow these changes in order to extract the most benefits for everyone.
